# Supplementary material for: Interaction-disorder-driven characteristic momentum in graphene, approach of multi-body distribution functions
Source: Sci Rep. 2019 Mar 6;9:3624. doi: 10.1038/s41598-019-39254-7 (PMC6403372; doi:10.1038/s41598-019-39254-7)
Supplement: Supplementary file 1 — Interaction-disorder-driven characteristic momentum in graphene, approach of multi-body distribution functions [file 41598_2019_39254_MOESM1_ESM.pdf]

Interaction-disorder-driven characteristic momentum in graphene, approach of multi-body distribution functions

M. N. Najafi[1,*]

[1]Department of Physics, University of Mohaghegh Ardabili, P.O. Box 179, Ardabil, Iran

ABSTRACT

1 Appendix1: Diagrammatic analysis of perturbative disorder in Thomas-Fermi-Dirac theory

In this appendix we analyze the disorder averaging and its diagrammatic interpretation. A complexity of the graphene system arises from the fact that the potentials are long-range for this system and the short-range techniques for treating the impurities[1] are not applicable. Let us rewrite the equation [7] for $\mu=0$ as follows:

$$n(\mathbf{r})=r_s^2\text{sgn}(n)\left[\frac{1}{2}\int d\mathbf{r}'\frac{n(\mathbf{r}')}{|\mathbf{r}-\mathbf{r}'|}+V_{xc}[n]+V_D\right]^2 \tag{1}$$

To obtain the sole effect of disorder and avoid unnecessary complexities, let us drop V_{xc}, and also ignore the effects of higher orders, i.e. $V_D^2\equiv 0$. By doing so, we obtain:

$$\begin{aligned}&n(\mathbf{r})|_{V_{xc}\equiv 0,V_D^2\equiv 0}=r_s^2\text{sgn}(n)\times\\&\left[\frac{1}{4}\int d\mathbf{r}_1 d\mathbf{r}_2\frac{n(\mathbf{r}_1)n(\mathbf{r}_2)}{|\mathbf{r}-\mathbf{r}_1||\mathbf{r}-\mathbf{r}_2|}+V_D(\mathbf{r})\int d\mathbf{r}_1\frac{n(\mathbf{r}_1)}{|\mathbf{r}-\mathbf{r}_1|}\right]\end{aligned} \tag{2}$$

Therefore, the Thomas-Fermi-Dirac relation can be cast to:

$$\begin{aligned}&\langle n(\mathbf{r})n(\mathbf{r}')\rangle=r_s^4\left(f_1(\mathbf{r},\mathbf{r}')+f_2(\mathbf{r},\mathbf{r}')+f_3(\mathbf{r},\mathbf{r}')\right)\\&f_1\equiv\frac{1}{16}\int d\mathbf{r}_1 d\mathbf{r}_2 d\mathbf{r}'_1 d\mathbf{r}'_2\frac{\langle\text{sgn}(n)\text{sgn}(n')n(\mathbf{r}_1)n(\mathbf{r}_2)n(\mathbf{r}'_1)n(\mathbf{r}'_2)\rangle}{|\mathbf{r}-\mathbf{r}_1||\mathbf{r}-\mathbf{r}_2||\mathbf{r}'-\mathbf{r}'_1||\mathbf{r}'-\mathbf{r}'_2|}\\&f_2\equiv\frac{1}{4}\int d\mathbf{r}_1 d\mathbf{r}_2\frac{\langle\text{sgn}(n)\text{sgn}(n')n(\mathbf{r}_1)n(\mathbf{r}_2)n(\mathbf{r}'_1)V_D(\mathbf{r}')\rangle}{|\mathbf{r}-\mathbf{r}_1||\mathbf{r}-\mathbf{r}_2||\mathbf{r}'-\mathbf{r}'_1|}+\mathbf{r}\leftrightarrow\mathbf{r}'\\&f_3\equiv\int d\mathbf{r}_1 d\mathbf{r}'_1\frac{\langle\text{sgn}(n)\text{sgn}(n')n(\mathbf{r}_1)n(\mathbf{r}'_1)V_D(\mathbf{r})V_D(\mathbf{r}')\rangle}{|\mathbf{r}-\mathbf{r}_1||\mathbf{r}'-\mathbf{r}'_1|}\end{aligned} \tag{3}$$

Noting that the $n(\mathbf{r})$ is a nonanticipating function, and V_D is a obtained from a white noise, we conclude that f_2 is related to $\langle\text{sgn}(n)\text{sgn}(n')n(\mathbf{r}_1)n(\mathbf{r}_2)n(\mathbf{r}'_1)\rangle\langle V_D(\mathbf{r}')\rangle$ which is manifestly zero. Also the first term (f_1) is higher order and is independent of disorder in the leading term. Therefore we consider only the effect of f_3. Now let us develop a diagrammatic representation of this term. Attributing a wavy line for the Coulomb interaction and a crossed circle for the external disorder, which have been defined in Fig. 1, one can draw some diagrams for the Thomas-Fermi-Dirac equations.

In the Fig. 2 we have shown the diagrammatic representation of the TFD energy, i.e. Eq. [4]. In this expansion, the first term is the Hartree terms, and the second term is the external interaction (the second term in the third line), and the third term represents the exchange-correlation term in this equation. Note that the exchange-correlation term is local in this expansion, as expected in the TFD theory. The quantity of interest in this paper is $G(\mathbf{r},\mathbf{r}')$, which has been shown in Fig. 3, whose right hand side represents f_3 (the first term). The higher order terms are the higher order iterations of the first term. It is worthy to note that in this expansion, only the effects of the disorder have been shown, and the other terms have been neglected. $G(\mathbf{r})$

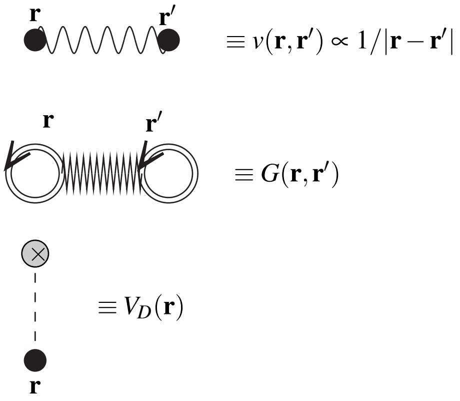

Figure 1. The diagrammatic representation of the interaction lines in the TFD theory.

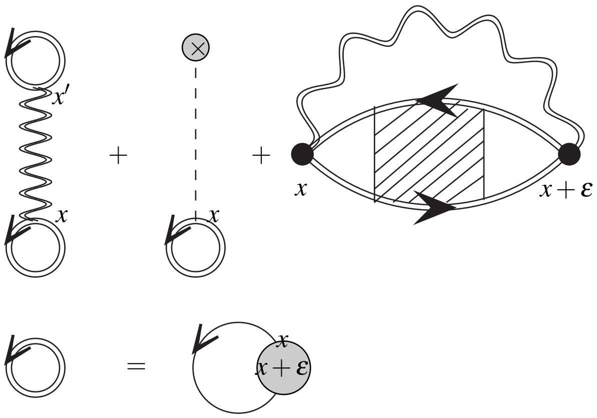

Figure 2. The diagrammatic representation of the TFD energy in the Eq. [4]. The second line shows the diagrammatic expansion of the full density $n(\mathbf{r})$. The gray circle shows the full electronic propagator which has become localized due to coarse graining. The local corrections of the graphs have been shown by diagrams which have been extended between x and $x+\varepsilon$.

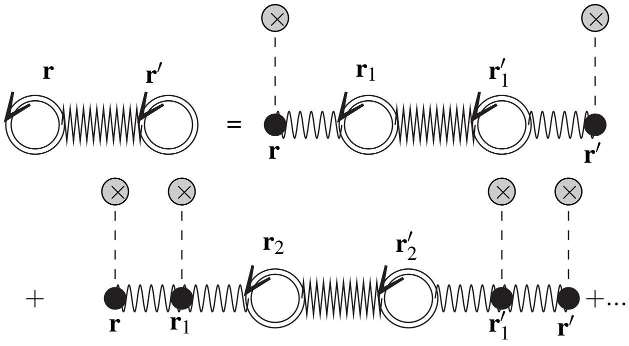

Figure 3. The diagrammatic representation of Eq 2.

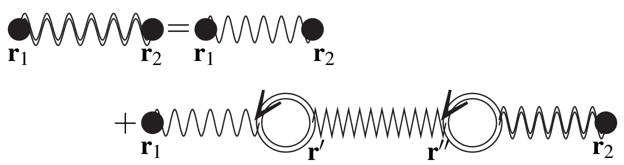

Figure 4. The diagrammatic expansion of the full Coulomb interaction line corresponding to the Eq. 4, with the mediator $G(\mathbf{r},\mathbf{r}')$.

has a non-trivial effect on the energy expectation value via[2]:

$$\begin{aligned}\left\langle\langle\Omega|\hat{V}|\Omega\rangle\right\rangle = \frac{1}{2}\int d^2\mathbf{r}d^2\mathbf{r}'V(\mathbf{r}-\mathbf{r}')\left[i\hbar\Pi(\mathbf{r},t;\mathbf{r}',t)_{\text{connected}}\right.\\ \left.+G(\mathbf{r},\mathbf{r}')-\delta\left(\mathbf{r}-\mathbf{r}'\right)n(\mathbf{r})\right]\end{aligned} \tag{4}$$

in which $\Pi(\mathbf{r},t;\mathbf{r}',t)_{\text{connected}}$ is the second term in Fig. [1] which also contains the disorder interaction lines[1]. The effect of the second term ($G(\mathbf{r})$) on the ground state energy is analyzed in this appendix in the Thomas-Fermi-Dirac (TFD) theory. This theory is a coarse-grained approximation for an electronic system, so that all the contributions are localized in a region in the close vicinity of the original spatial point, except the Hartree term, as well as the disorder term which are non-local terms. The Hartree term arises from the second term in the right hand side of Eq. 4. These have been shown in the Fig. 2 in which the first term is the Hartree term, the second one is the external disorder interaction and the last term shows the other terms which are local in the coarse-grained system (to show this, we used $x+\varepsilon$ to mention the close vicinity of x). The double (coulomb and electronic) lines show the full propagators. The double-line circles show the full electronic density which are the solution of the TFD equations and have been shown in Fig. 4. Full lines have simultaneously the disorder and coulomb interaction lines.

Now let us consider the screening of the potential in one-loop level. With an appropriate contractions in the first order perturbation, we have in the real space:

$$\begin{aligned}&v(\mathbf{r}_1-\mathbf{r}_2) = v^0(\mathbf{r}_1-\mathbf{r}_2)+\\ &\frac{i}{\hbar}\int\int d^2\mathbf{r}'d^2\mathbf{r}''v^0_{\mathbf{r}_1,\mathbf{r}'}v^0_{\mathbf{r}'',\mathbf{r}_2}G(\mathbf{r}'-\mathbf{r}'')+\text{higher order terms.}\end{aligned} \tag{5}$$

in which $v^0_{\mathbf{r}_1,\mathbf{r}_2}\equiv v^0(\mathbf{r}_1-\mathbf{r}_2)$. In the coarse-grained version, this term (and also higher order terms containing $G(\mathbf{r},\mathbf{r}')$) survives and plays an important role. In the third line of the Fig. 4 we have shown the infinite series of the above equation, in which the most left bare coulomb line is replaced by the full line. By taking Fourier transform of this series, and defining $G(q)=\int d^2\mathbf{r}e^{iq.\mathbf{r}}G(\mathbf{r})$, we reach to the following equation:

$$v(q) = \frac{v^0_q}{\varepsilon(q,\omega)} \tag{6}$$

in which the dielectric function is determined to be $\varepsilon(q,\omega)=1+\frac{i}{\hbar}v^0_qG(q)\delta(\omega)$. In the real systems, the time scales up to the time required for a photon to pass through the sample can be interpreted a instantaneous, i.e. $\delta(\omega)\sim\tau_{\text{ch}}\equiv\frac{d}{c}$. Using this fact and the relation [30] and the fact that $\varepsilon(G_q=0)=\kappa_S$, one can show that:

$$\begin{aligned}\varepsilon(q,\omega) &\approx \kappa_S + i\frac{\gamma_0\kappa_S}{dr_s^3}\left(\frac{v_F}{c}\right)\frac{\tilde{G}(\tilde{q})}{q}\\ &= \kappa_S\left(1+i\frac{\tilde{q}_0}{\tilde{q}}\tilde{G}(\tilde{q})\right)\end{aligned} \tag{7}$$

in which $\tilde{q}_0\equiv\frac{\gamma}{n_id^2r_s^5}\left(\frac{v_F}{c}\right)$, $\gamma_0\equiv 128$ and $\gamma\equiv 512\sqrt{2/\pi}$ and $\tilde{q}\equiv\frac{n_i\zeta'}{\alpha}q$ as defined in the SEC. [3]. For a system with $d^2n_i\sim 1$ and $r_s=0.8$, $\tilde{q}_0\sim 23.5$. One may concern about the limits $n_i\to 0$ or $r_s\to 0$ for which $\tilde{q}_0\to\infty$. This actually is not a problem since for these quantities $\tilde{q}\to\infty$ for which $\tilde{G}$ is zero. In fact in this limits the imaginary part of $\varepsilon(q,\omega)$ vanishes (as is seen in the first

line of the above equation) as expected. Therefore the normalized screened potential, defined by $V_q \equiv \frac{\alpha}{4\pi e^2 n_i \zeta'} v_q = \frac{d r_s^2 n_i}{16\sqrt{2\pi} e^2} v_q$ (so that $V_q^0 = \tilde{q}^{-1}$) takes the following form:

$$\kappa_S \mathrm{Re}[V_q] = \frac{V_q^0}{1+\frac{\tilde{q}_0^2}{\tilde{q}^2}\tilde{G}(\tilde{q})^2} \tag{8}$$

This result should be compared with the relation $\varepsilon(q) = \varepsilon_0 - \frac{qe^2}{2\omega_p(q)} \ln\left(\frac{2|\mu|-\omega_p(q)}{2|\mu|+\omega_p(q)}\right)$ which was obtained by Shung that yields the change of the dielectric function and leads to the relation[3]:

$$\frac{v_0(q)}{\varepsilon(q,0)} = \frac{1}{\varepsilon_0}\frac{2\pi e^2}{q+q_{\mathrm{TF}}} \tag{9}$$

in which $q_{\mathrm{TF}} \equiv 4\pi e^2 k_F / v_F \varepsilon_0$. In our case one can easily show that:

$$\begin{aligned} \frac{v_{\mathrm{sc}}(r)}{4\pi e^2} &= \frac{1}{4\pi e^2}\int d^2q \frac{v_0(q)}{\varepsilon(q,\omega)} e^{iq.r} \\ &= \frac{\sqrt{\pi} d n_i^2 r_s^2}{4\sqrt{2}\kappa_S} f_1(R) \\ \frac{\delta n(r)}{4\pi e^2} &= \frac{1}{4\pi e^2}\int d^2q \frac{v_0(q)G(q)}{\varepsilon(q,\omega)} e^{iq.r} \\ &= \frac{8\sqrt{\pi}}{\sqrt{2}\kappa_S d}\left(\frac{n_i}{r_s}\right)^2 f_2(R) \end{aligned} \tag{10}$$

in which $v_{\mathrm{sc}}(r)$ is the screened potential which is obtained from $\varepsilon(q,\omega)$ and:

$$\begin{aligned} f_1(R) &\equiv 2\pi \int_0^\infty \frac{J_0(\tilde{q}R)}{1+\left(\frac{\tilde{q}_0}{\tilde{q}}\tilde{G}(\tilde{q})\right)^2} d\tilde{q} \\ f_2(R) &\equiv 2\pi \int_0^\infty \frac{\tilde{G}(\tilde{q}) J_0(\tilde{q}R)}{1+\left(\frac{\tilde{q}_0}{\tilde{q}}\tilde{G}(\tilde{q})\right)^2} d\tilde{q} \end{aligned} \tag{11}$$

It is notable that in the limit $n_i \to 0$ one obtains the trivial result $v_{\mathrm{sc}}(r) \to v_0(r)$.

2 Appendix2

In this short appendix we concentrate on calculating $\mathrm{d}\chi_n(0) \equiv \nabla\chi_n(0).\mathrm{d}\mathbf{r}$ and $(\mathrm{d}\chi_\rho(d))^2 \equiv (\nabla\chi_\rho(d).\mathrm{d}\mathbf{r})^2$. Writing $\mathrm{d}\chi_\rho(d)$ as $\int d^2\mathbf{r}'\rho(\mathbf{r}')\left[\left(|\mathbf{r}+\mathrm{d}\mathbf{r}-\mathbf{r}'|+d^2\right)^{-1/2} - \left(|\mathbf{r}-\mathbf{r}'|+d^2\right)^{-1/2}\right]$ we have:

$$\begin{aligned} &\left\langle(\mathrm{d}\chi_\rho)^2\right\rangle = \int d^2\mathbf{r}' d^2\mathbf{r}'' \\ &\left[(|\mathbf{r}-\mathbf{r}'|^2+d^2)(|\mathbf{r}-\mathbf{r}''|^2+d^2)\right]^{-\frac{1}{2}} \times \\ &\left\langle\left(\rho(\mathbf{r}'+\mathrm{d}\mathbf{r})-\rho(\mathbf{r}')\right)\left(\rho(\mathbf{r}''+d\mathbf{r})-\rho(\mathbf{r}'')\right)\right\rangle \end{aligned} \tag{12}$$

By expanding the integrand in terms of $\mathrm{d}\mathbf{r}$, i.e. $\left(|\mathbf{r}-\mathbf{r}'-\mathrm{d}\mathbf{r}|^2+d^2\right)^{-1/2} \simeq \left(|\mathbf{r}-\mathbf{r}'|^2+d^2\right)^{-1/2} + \mathrm{d}\mathbf{r}.\nabla\left(|\mathbf{r}-\mathbf{r}'|^2+d^2\right)^{-1/2}$, we obtain

$$\left\langle(\mathrm{d}\chi_\rho)^2\right\rangle = 2(n_i d)^2 \int d^2\mathbf{r}' \frac{(\mathbf{r}-\mathbf{r}').\mathrm{d}\mathbf{r}}{(|\mathbf{r}-\mathbf{r}'|^2+d^2)^2} \tag{13}$$

By replacing $\cos^2\theta \equiv \frac{1}{2}$, we see that $\left\langle(\mathrm{d}\chi_\rho)^2\right\rangle = \frac{\pi d n_i^2}{2\sqrt{2}}\mathrm{d}r$. Note that $\mathrm{d}\chi_n(0) \simeq \frac{1}{\sqrt{2}} G_n \mathrm{d}r$ in which $G_n = \int d^2\mathbf{r}' \frac{n(\mathbf{r}')}{|\mathbf{r}-\mathbf{r}'|^2}$.

3 Appendix3

This appendix has been devoted to the calculation of functional derivative of P. Our functional derivatives are a bit different from the common definitions which needs clarification. Let us consider the integral

$$\mathrm{d}\langle f_{\mathbf{r}_0}\rangle = \int d^2\mathbf{r} \int D[n]\mathrm{d}X(n(r))P(\{n\})\,\partial_n f_{\mathbf{r}_0}(n(r)) \tag{14}$$

To define the derivatives we mesh the system by squares of sizes $d \times d$, so that the above integral becomes:

$$\begin{aligned}\mathrm{d}\langle f_{\mathbf{r}_0}\rangle =& \sum_i d^2 \int dn_0 dn_1 dn_2 ... dn_i ... P(n_0, n_1, n_2, ..., n_i, ...)\times \\ & \mathrm{d}X(n_i)\partial_{n_i} f(n_i, n_0) \\ =& -d^2 \sum_i \int dn_0 dn_1 dn_2 ... dn_i ... \\ & \partial_{n_i}\left[P(n_0, n_1, n_2, ..., n_i, ...)\mathrm{d}X(n_i)\right] f(n_i, n_0)\end{aligned} \tag{15}$$

The second equality was obtained using integration by parts. If we perform the integration of the above equation over $D[n]' \equiv dn_1 ... dn_{i-1} dn_{i+1} ...$, the equation governing $f(\mathbf{r}_0, \mathbf{r}) = \langle n(\mathbf{r}_0, n(\mathbf{r}))\rangle$ presented in SEC. **??** is obtained, noting that:

$$\begin{aligned}P(n, \mathbf{r}; n', \mathbf{r}_0) &= \int D[n]' P(\{n\}) \\ &= \int \prod_{j\neq 0, i} dn_j P(n_0, n_1, ..., n_i, ...)\end{aligned} \tag{16}$$

in which $n_j \equiv n_j(\mathbf{r}_j)$ and $\mathbf{r} \equiv \mathbf{r}_i$. On the other hand, as explained in the text, we consider $P(\{n\})$ to be of the form $\exp\left[-\frac{1}{d^2}\int d^2\mathbf{r}' d^2\mathbf{r}'' n(\mathbf{r}')n(\mathbf{r}'')H(\mathbf{r}', \mathbf{r}'')/|\mathbf{r}' - \mathbf{r}''|\right]$. Therefore we have ($H_{i,j} \equiv H(n(\mathbf{r}_i), n(\mathbf{r}_j))$, $r_{i,j} \equiv |\mathbf{r}_i - \mathbf{r}_j|$ and noting that $\int\int d^{\mathbf{r}} d^2\mathbf{r}' \to d^4 \sum i, j$):

$$\begin{aligned}P &\sim \exp\left[-\frac{1}{d^2}\sum_{i,j} d^4 \frac{n_i n_j}{r_{i,j}} H_{i,j}\right] \\ &= \prod_{i\neq j} \exp\left[-d^2 \frac{n_i n_j}{r_{i,j}} H_{i,j}\right]\end{aligned} \tag{17}$$

which yields

$$\begin{aligned}\partial_{n_i} \ln(P(\{n\})) &= -d^2 \sum_j \left[\frac{n_j}{r_{i,j}}(2 + n_i \partial_{n_i}) H_{i,j}\right] \\ \text{in continuum limit} &\longrightarrow -\int d^2\mathbf{r}' \frac{n(\mathbf{r}')}{|\mathbf{r} - \mathbf{r}'|}(2 + n(\mathbf{r}')\partial_{n(\mathbf{r})}) \\ &\times H(n(\mathbf{r}), n(\mathbf{r}'))\end{aligned} \tag{18}$$

which coincides the calculations in the main text.

References

1. Sadovskii, M. V. *Diagrammatics: lectures on selected problems in condensed matter theory* (World Scientific, 2006).

2. Fetter, A. L. & Walecka, J. D. *Quantum theory of many-particle systems* (Courier Corporation, 2012).

3. Shung, K. W. K. Dielectric function, screening, and plasmons in two-dimensional graphene. *Phys. Rev. B* **34**, 979 (1986).
